# Facilitators and barriers to medication self-management for patients with multiple long-term conditions transitioning from hospital to home

**DOI:** 10.1016/j.rcsop.2025.100598

**Published:** 2025-03-29

**Authors:** Malin Olsen Syversen, Mikas Glatkauskas, Liv Mathiesen, Marianne Lea, Berit Gallefoss Denstad, Karin Svensberg

**Affiliations:** a Department of Pharmacy, Section for Pharmacology and Pharmaceutical Biosciences, University of Oslo, Oslo, Norway; b Department of Pharmaceutical Services, Oslo Hospital Pharmacy, Hospital Pharmacies Enterprise, South-Eastern Norway, Oslo, Norway; c Users representative, Lillehammer, Norway; d Department of Pharmacy, Uppsala University, Uppsala, Sweden

**Keywords:** Self-management, Medication therapy management, Hospital to home transition, Multiple chronic conditions, Facilitators and barriers

## Abstract

**Background:**

Being a patient with multiple long-term conditions (MLTCs) often entails a need for complex medication treatment, which poses a challenge to medication self-management. Medication self-management during transition of care is often hindered by challenges such as inadequate communication, which increases the risk of medication errors and adverse outcomes.

**Aim:**

Identify facilitators and barriers to medication self-management for patients with MLTCs transitioning from hospital to home.

**Methods:**

Semi-structured interviews were conducted in patient's homes 1–2 weeks after hospital discharge. Interviews were transcribed and analysed by qualitative deductive content analysis using the Taxonomy of Every Day Self-management Strategies (TEDSS) framework. The data collection continued until enough information power and meaning saturation was reached.

**Results:**

Twenty-one patients and three next of kin participated. Numerous facilitators and barriers to medication self-management were identified within all seven TEDSS domains, which varied extensively between individuals. Resource and process strategies were the most frequently discussed domains, while health behaviour and social interaction strategies were less frequently discussed. Key facilitators identified were access to resources that support medication self-management and knowing the medication's purpose. Key barriers included patients perceiving medications as burdensome or not recognising the importance of their medications.

**Conclusions:**

This study highlights the complex and wide spectre of facilitators and barriers to medication self-management for patients with MLTCs transitioning from hospital to home. In clinical practice, patients' medication self-management could be supported through a holistic approach adapted to the individual patient's daily life, including improved care coordination and patient empowerment.

## Highlights

• Provides facilitators and barriers to medication self-management for patients with MLTCs.
• Numerous and various strategies affecting medication self-management were identified.
• Heavy focus on facilitators and barriers regarding resources and processes.

## 1 Background

Being a patient with multiple long-term conditions (MLTCs) often entails a need for complex medication treatment. Consequently, MLTCs can significantly impact quality of life, escalate medical expenses, increase medical demands, and affect individuals' ability to take care of themselves.^1^^,^^2^ These patients use several medications and are often at higher risk for readmissions and hospitalisations, which lead to multiple transitions of care.^3^^,^^4^ In an often fragmented healthcare system, care transitions require increased coordination as inadequate communication threatens patient safety by the risk of losing critical medical information, leading to an increased risk of numerous adverse outcomes, including medical errors.^5^^,^^6^ Hence, it is unsurprising that patients with MLTCs face hurdles within the healthcare system and that transitions between healthcare levels make patients vulnerable.^5^

Self-management, commonly defined in a healthcare context as tasks individuals perform to live well with one or more long-term conditions, can be a crucial factor for effective care transitions.^7^^,^^8^ Self-management has gained increased attention, but it is rarely a systematic nor integrated part of patient care.^7^^,^^9^ Studies have shown that the discharge process is not always tailored to prepare the patient for post-discharge self-management.^10^^,^^11^ As a consequence, patients might face challenges and continue to feel unprepared for self-management after hospital discharge.^12^^,^^13^ High capacity of self-management has been associated with decreased healthcare usage^8^ and improved health outcomes.^14^ A patient-centred focus should, therefore, be incorporated into healthcare services to increase patients' capacity for self-management and to customise self-management to individual patient preferences and goals.

**Fig. 1:**
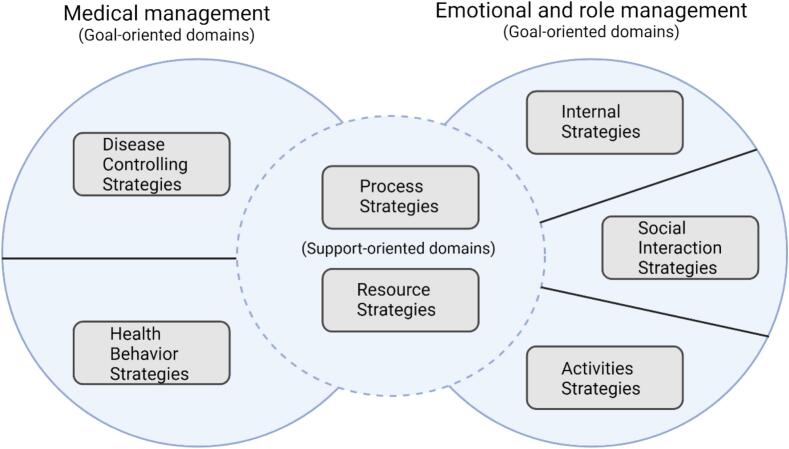
An illustration of the five goal-oriented domains (disease controlling, health behaviour, internal, social interaction, and activities) and the two support-oriented domains (process and resource) in the Taxonomy of Every Day Self-management Strategies (TEDSS) framework in relation to the three components of self-management (medical, emotional and role). Inspired by A. Audulv et al.^18^ Created in BioRender.com. BioRender.com/g95t178.

Medication management is an important component of self-management for patients with MLTCs, and various frameworks to map components of self-management have emerged.^9^ However, the majority of medication management frameworks focus on adherence or specific diseases^9^^,^^15^^,^^16^ and not on the core components of self-management.^17^ The Taxonomy of Every Day Self-management Strategies (TEDSS) framework provides a patient-centred approach to understanding how patients self-manage all aspects of everyday life with one or more long-term conditions by integrating all three components of self-management, i.e., medical, role, and emotional.^18^ These three components are divided into seven broad domains: five goal-oriented domains (disease controlling, health behaviour, internal, social interaction, and activities) and two support-oriented domains (process and resource), see Fig. 1. Within the seven domains, there are 26 subdomains. The TEDSS framework has not been developed to capture medication self-management alone but has previously been used for this in another population.^19^ Due to their diverse needs and complex medication regimens, exploring medication self-management is especially important for patients with MLTCs.

Self-management has been explored in different populations and settings,^9^^,^^13^^,^^20^ but there are few descriptions of facilitators and barriers to medication self-management in the transition between healthcare levels for patients with MLTCs. A qualitative approach is essential to capture the depth and complexity of patients' experiences with medication self-management by uncovering insights that may not emerge through pre-defined survey questions. This approach allows for a more contextualised understanding, which is crucial for identifying actionable facilitators and barriers in real-world settings. Therefore, the aim of this study is to identify facilitators and barriers to medication self-management for patients with MLTCs transitioning from hospital to home.

## 2 Method

### 2.1 Study design and setting

This qualitative study is part of a larger research project exploring medication use across healthcare levels. Semi-structured interviews conducted with patients and their next of kin following hospital discharge were deemed the most appropriate method to capture the patient's perspective.^21^ This study is reported following the consolidating criteria for reporting qualitative studies (COREQ) checklist.^22^

In Norway, patients do not bring their own medications to the hospital but receive medications from the hospital during their stay. After hospital discharge, patients responsible for their own medication management will normally go to a pharmacy to get their prescribed medications.^23^^,^^24^ According to Norwegian regulations, an updated and reconciled list of medications in use should, in agreement with the patient, always follow the patient when transitioning from hospital to home.^25^

### 2.2 Participants and recruitment

Patients ≥18 years were included close to the day of their planned discharge from an internal medicine ward and two geriatric wards at a university hospital in Norway from December 2022 to February 2024. To be included in the overall research project, patients needed a residential address in Oslo, had to normally reside at home, and manage their medications independently (or with limited assistance from home care nurses or next of kin). Patients were required to be discharged to their homes or short-term stay in nursing homes. They should have been prescribed at least four regular medications from a minimum of two therapy classes (Anatomical Therapeutic Chemical (ATC) at first level^26^). Additionally, patients should have at least two long-term conditions. Terminally ill patients, those isolated due to infections, and individuals with advanced cognitive decline as assessed by the treating physician were excluded. Previous study participation and inability to communicate in Norwegian or English were also reasons for ineligibility.

A subset of patients included in the large research project was purposively selected^27^ with regard to characteristics such as gender, age, ethnicity, education, number of medications, and help from next of kin/home care nurses or not, to participate in the semi-structured interviews. Next of kin were included at the time of the interview if they were involved in the patient's medication management after hospital discharge.

### 2.3 Data collection

An interview guide was developed based on the knowledge gap and discussions in the research team, with specific input from the user representative. The following topics were explored in the interviews: self-management, medication information, and shared decision-making. The interview guide (Supplemental file 1) contained questions and probes, and these were continuously evaluated and consequently adjusted two times (after interview number four and nine) to gain more insight and a broader understanding of specific themes during the data collection period. The interviewers (authors MOS and MG) received thorough training in interviewing methodology from an experienced qualitative researcher (author KS) prior to conducting the interviews, e.g., how to ask questions and get participants to elaborate on these. In addition, regular meetings within the project group were held between interviews to discuss, exchange experiences, and ensure methodological consistency.

Patients were contacted by phone after discharge to schedule a time for the interview. The interviews were performed at the patient's home or at an office 1–2 weeks after discharge. Interviews took place mainly Monday to Friday. At first, a medication reconciliation (MR) was performed, and demographic variables were collected. Thereafter, the participant took part in an in-depth, semi-structured interview. Both the MR and the interviews were audio recorded. In addition, field notes were taken under or immediately after the interviews and included in the analysis.

The data collection continued until we had enough information power^28^ and achieved meaning saturation.^29^ Each interview was compared with previous ones to identify similarities and differences, as well as to evaluate how the content of the categories in TEDSS evolved during the analysis of each new interview. Saturation was continuously evaluated during the data collection and analysis process, meaning that no new insights were obtained in the last interviews. Also, to enrich the data, we selected some patients for follow-up interviews after an additional 3–4 weeks.

### 2.4 Pre-understanding

Authors MOS and MG are PhD-students and have master's in pharmacy, within the field of clinical pharmacy and pharmacology, respectively. The research team comprises pharmacists with a PhD (authors LM, ML, and KS*)* with experience in research within clinical and social pharmacy, as well as clinical experience, and a user representative (author BGD).

### 2.5 Data analysis

Data analysis followed qualitative deductive content analysis^30^ guided by the TEDSS framework.^18^ The TEDSS framework was used to explore medication self-management discussions among participants focusing on the transition from hospital to home. In addition, TEDSS subdomain data were sorted into positive (+) or negative (−) categories to identify facilitators and barriers to medication self-management. Audio files were uploaded to NVivo v.12 for transcription and further analysis. The MR was included in the analysis since the information obtained contributed to a broader understanding of the interview data. First, four authors (MOS, LM, ML, and KS) independently coded two transcripts according to the TEDSS framework. Then, these authors collectively reviewed the coding to ensure a shared understanding of the framework. In cases of differences in coding, we discussed them until an agreement was reached and further descriptions were added to the codebook (Supplemental file 2). Then, author MOS independently coded five more transcripts before another joint coding meeting. As no substantial changes were made, author MOS coded the rest of the transcripts. Author MOS condensed the data from the domains and subdomains into analytical texts, which were discussed. Final analytical texts were then compared to the original interview data.

### 2.6 Ethical considerations

All participants gave written informed consent before inclusion. The participants could withdraw their consent at any time, without any justification. Following consent, the data collectors were given access to the patient's hospital records. The project was approved by the Regional Committee for Medical and Health Research Ethics (Ref No 420920/REK south-eastern C), Sikt (Ref No 919319), and the Privacy Ombudsman at Oslo University Hospital. After the data collection, participants were offered a small gift (5€). The data collectors were not working at the hospital wards. Participation in the study did not pose any risk or harm to the participants beyond the time spent. All data were stored and managed on a secure research server at the University of Oslo.

## 3 Results

A total of 21 patients and three next of kin participated in this study. The patients' characteristics are presented in Table 1. All interviews except one were conducted in the patient's home, and the last was conducted at an office. The three next of kin included two women who were partners and one woman who was the daughter of a patient.

**Table 1:** Patient characteristics.

Characteristic (21 patients)	
Age in years, median (range)	72 (22–94)
Gender, number (percent)	
Women	11 (52 %)
Men	10 (48 %)
Number of patients living alone (percent)	13 (62 %)
Area of origin, number (percent)	
Norwegian	18 (86 %)
Other (Scandinavian, European, African)	3 (14 %)
Education, number (percent)	
No university degree	12 (57 %)
University degree	9 (43 %)
No health education	18 (86 %)
Health education	3 (14 %)
Assistance with medication administration post-discharge, number (percent)	
Manage themselves	13 (62 %)
Manage themselves with help from home care nurse	5 (24 %)
Manage themselves with help from next of kin	2 (9 %)
Manage themselves with help from home care nurse and next of kin	1 (5 %)
Receiving multidose drug dispensing system, number (percent)	3 (14 %)
Number of medications in the discharge summary, median (range)	9 (5–21)
Number of medications in use after medication reconciliation, median (range)	10 (5–21)
Number of patients with one or more medication discrepancies (percent)⁎	18 (86 %)
Number of long-term conditions at discharge, median (range)	6 (2−13)

⁎ Between the discharge summary and medication use after hospital discharge.

The interviews averaged 59 min (±16) (including MR 95 min (±22)) and took place on average 11 days (±4.3) post-hospital discharge. Three individuals participated in additional follow-up interviews averaging 49 min (±6), at an average of 34 days (±2.5) after the initial interview.

**Fig. 2:**
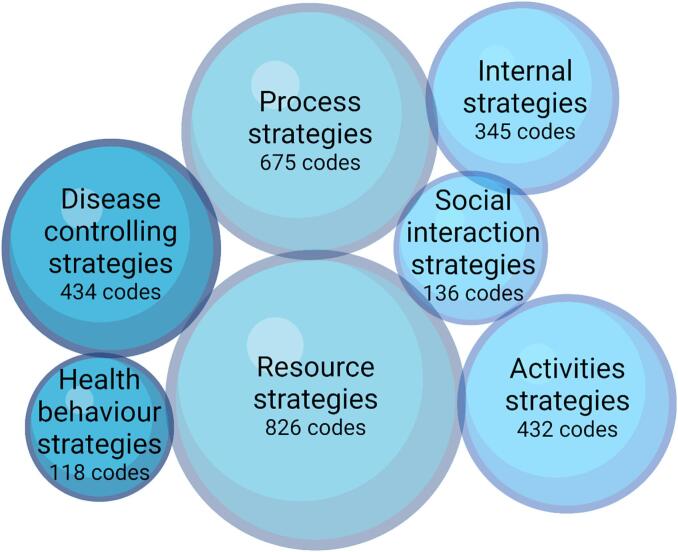
Number of codes in each of the Taxonomy of Every Day Self-management Strategies (TEDSS) domains. Created in BioRender.com. BioRender.com/a64b058.

All domains in the TEDSS framework were covered in the interviews, and only one subdomain was not discussed at all (*seeking comfort in faith and spirituality*). The participants discussed self-management to control their medications, starting all the way from the hospital stay until the interview. The results are presented according to the five goal-oriented and the two support-oriented domains (see Fig. 1), and the presented data focuses on medication self-management in the transition from hospital to home. Fig. 2 shows the number of codes in each of the TEDSS domains to illustrate what was mostly discussed. Facilitators for medication self-management were more frequently described than barriers by the participants. Examples of facilitators and barriers for each subdomain are presented in Supplemental file 3. The subdomain *seeking and managing health/social-care needs* within the resource strategies domain were the most discussed, followed by the subdomain *information seeking* in the process strategies domain. A more detailed description of each domain and subdomain can be found in Supplemental file 2.

### 3.1 Medical management

#### 3.1.1 Disease controlling strategies

The patients talked about prescribed and over the counter medications, however, to a limited degree, they discussed non-medication alternatives. Patients described adhering to their prescribed medication regimen despite the MR revealing that almost all patients had one or more medication discrepancies between the discharge summary and actual use. Several were supposed to start using new medications or supplements, but some were unaware of this and had not used them. Some patients did not take any specific action to self-manage their medications after returning home and instead preferred not to use them, as they associated medications with illness. However, most patients acknowledged the importance of taking their medications. Most patients said that taking their medications as prescribed affected their quality of life positively, while a few did not see any link between quality of life and medications. Some used medication taking as a training situation, e.g., to gain back function after a stroke by opening medication packages to increase hand function. On occasions, patients had not taken their medications as prescribed after hospital discharge, for example, because of personal preference or the tablet popped out of the pill organiser and was lost. Some described how they had to split tablets to get the right dosage to complete treatments after discharge, which acted as a barrier to medication self-management.

Self-management facilitators and barriers to control symptoms and monitor side effects after hospital discharge were frequently described. Most patients facilitated taking medications by having sufficient medications available, but some also described not having necessary medications at home as a barrier to prevent symptoms, such as pain or anxiety, resulting in insufficient relief. Self-management of pain was frequently discussed, and some talked about facilitators to reduce and discontinue their medication in a short time perspective. Some patients used on demand medications diligently or even more than prescribed. Others preferred not using medications to prevent symptoms or did not understand the reason for using them, impacting their medication self-management ability. These patients used their on demand medications as little as possible, postponed the administration as long as possible, or preferred to wear the symptoms off, e.g., sleeping away pain.

*I don't like taking so much medication, so yes, you must put up with a little pain. (♂, P16).*

Some patients described self-managing complications or controlling side effects from medications prescribed at the hospital by using on demand medications as a facilitator, e.g. medications against constipation caused by opioids. One patient experienced a side effect that worsened after hospital discharge until it became unbearable. Subsequently, the patient contacted the general practitioner (GP) and was prescribed another medication to facilitate self-management of the side effect. Others just coped with side effects and waited to see if they disappeared. Several patients also talked about medications they decided to use despite describing barriers to getting the medications out of the package or experiencing side effects. One of these wished to stop using a medication but continued anyway.

*What is both with this (medication) and why I would like to get rid of this is that it destroys, it is unpleasant to drink, not unpleasant, but it seems to me it is goofy to drink mineral water and beer (together with the medication) because of the carbonation. (♂, P11).*

Almost all patients self-managed by using supplements such as vitamins, minerals, probiotics, or herbal remedies to supplement their medications after discharge. Many talked about being sceptical of or having no belief in some supplements as barriers but had decided to give them a try anyway. Others wanted to adhere to the physicians' prescriptions and not use anything in addition. However, supplements seemed less important to incorporate into daily routines than medications since the patients often took supplements when they remembered. On the other hand, for some, it was important to take supplements regularly, e.g., to keep the bone structure intact. The knowledge about supplements acted as a facilitator for these patients to manage their conditions and medication use.

#### 3.1.2 Health behaviour strategies

Diet was discussed in almost all interviews. In general, patients said it was important to know if their medications interacted with foods and drinks as this could impact their medication self-management ability. Patients had changed or moderated their diet to avoid using medications after hospital discharge, e.g., prunes or yoghurt to manage constipation or dairy products to strengthen bone structure. Newly diagnosed diabetes patients described adjusting on demand insulin according to food intake by measuring blood sugar levels often and avoiding certain foods to minimise on demand insulin needs as facilitators to medication self-management. One patient was unsure how the insulin needs would be after hospital discharge due to differences between hospital food and diet at home, in addition to remembering to administer the insulin before every meal.

*Now I think it (the insulin) kind of interrupts a lot of things, and it bothers me a bit, which I must remember, like taking it every day before meals. (..) I hope soon it will just be a habit and I won't have to think about it that much. (♀, P3).*

Mental exercises were brought up by about half of the patients as a facilitator to self-manage their medications after discharge. Reading books, household chores, games, association membership with peers, and volunteering were among the facilitators to keep mentally fit to be able to self-manage medications, while others did not prioritise or see the benefits of mental exercise. One next of kin talked about doing more challenging things with the patient after the patient's hospital discharge to help the patient gain back mental status after a stroke and support medication self-management.

After hospital discharge, most patients did some kind of physical exercise within their level of functional ability to improve medication self-management, such as exercises at home or physiotherapy. Many described physical exercise as an important component to achieving their goals faster, such as coming back to normal daily activity or managing medications independently. Some also used exercise to stop or avoid medications. A newly diagnosed diabetes patient noticed that physical exercise affected blood sugar levels after being discharged from the hospital, leading her to adjust the insulin dose accordingly. An example of a facilitator for physical exercise was using an inhaler prior to activity. Barriers were lack of motivation or economy. Some patients refused physiotherapy because of the fees or insufficient medications to limit pain during physical exercise. One patient described receiving a painkiller before physiotherapy at the hospital and expressed a wish to continue this practice at home.

Some described that they had gotten less sleep at the hospital and that this influenced their sleep routines. For some, this returned to normal after hospital discharge, while for others, the trouble falling asleep continued and caused a need for sleeping pills. A few patients were prescribed antibiotics at hospital discharge, dosed four times daily, and carefully followed this routine, which acted as a barrier by shortening the night's sleep. Others facilitated to maintaining normal sleep hygiene after hospital discharge by having regular bedtimes or doing calming activities to avoid using sleep medications. Some described that their medication regimen, having to take medications at the same time every day, facilitated regular bedtime. One patient described taking the cortisone medication in the morning, being aware that this medication could negatively influence sleep if taken at nighttime.

### 3.2 Emotional and role management

#### 3.2.1 Internal strategies

Acceptance frequently appeared in the interviews, and most patients seemed to accept their conditions and use of new medications, often in addition to medications used prior to the hospital stay. They described having to accept conditions and medications to facilitate mastery. Many patients appreciated to control and have responsibility for their medications after hospital discharge. In this way, they gained knowledge about their own health and medications to be independent. Several even talked about medications as a safety and necessity to prevent deterioration or readmission. Despite this, some patients talked about avoiding involvement around medications or not being comfortable asking questions about new conditions or medications, just accepting the changes, and trusting the knowledge of their healthcare professionals (HCPs) as barriers for their ability to self-manage medication. In addition, several patients wished they did not need so many medications or considered them as a burden, which acted as barriers.

*I would rather not take (medications), but now I have to. I know that. (♀, P13).*

Some patients had come to terms with using a multidose drug dispensing system or receiving help from home care nurses after hospital discharge to facilitate medication self-management and avoid medication errors, despite some resignations. Others had refused help since they thought they could self-manage their medications alone. One who had accepted receiving help with administering medications and supplements was not aware of what was given and continued taking his supplements independently, i.e., taking double doses. Another had accepted help to fill the pill organiser due to frequent dose adjustments to facilitate self-management, but instead, experienced this as a barrier due to the loss of control.

*I know I agreed to this. I know then I won't do it myself (fill the pill organiser). That this might become so complicated that it makes sense to have it that way, but I hadn't thought it through, and that I then lost a bit of the overview. I suddenly became very interested in that. Now, I lost a bit of my self-control. (♂, P14).*

A few patients said they sometimes expressed feelings to relieve stress and feel better after hospital discharge, e.g., being sad about their situation. Some also expressed feelings by crying, sometimes due to pain, despite using painkillers. One patient had stopped smoking before hospital admission and used nicotine patches to relieve his symptoms. After discharge, the new situation made the patient sad about being homebound and alone, which made him seek comfort in smoking again. This patient planned to stop smoking and use nicotine patches again once his situation got better. To facilitate this, the patient sought new arenas for activity.

*I was mentally prepared, but still, you get a little. (..) I can get sad and upset. (I) can get a little wet in the eyes. It's happening right now. Here we are back to smoking, that's the consolation. So, I expect to get a little stronger at it. (..) I am cut off from several things, (such as) getting out. I sit in the doorway and look out. (♂, P11).*

Facilitators to control stress and negative emotions mostly discussed were avoiding thinking about problems, symptoms, or future risks by focusing on other thoughts or activities. Others used techniques such as mindfulness or relaxation listening to music, sometimes to avoid medications. Some patients adopted a mindset of not having any long-term conditions. This was facilitated by taking medications, thereby not having noticeable symptoms and being able to focus on coming back to valued activities.

*I am a hobby shooter. (..). And I've been doing that since I got out of the hospital. So, I don't feel that there are any limitations to that extent. I'm not trying to wear myself out completely, but to go and have the social part and to be allowed to train and shoot the way I do, I think that's important. Start sitting down and not doing things like that to wait and see if you get any better. No, then I think it's better to be active. Don't focus too much on what has happened. (♂, P16).*

Some described recognising and managing symptoms by contacting HCPs, knowing whom to contact, rather than postponing contact or having scheduled appointments as facilitators to medication self-management and control stress after hospital discharge. One patient had been at a follow-up appointment, which was described as a facilitator, giving renewed confidence to self-manage new medications and conditions. To avoid possible negative situations, some patients made proactive choices. A facilitator for medication self-management at home was not reading about side effects but rather focusing on knowing why they used the medication. Other patients talked about a wide spectre of stress and negative emotions related to medication self-management but did not seem to have any methods to control them.

Life post-discharge varied, but most patients described being positive and motivated to use medications as a facilitator to get better. Some patients were negative to changes, such as receiving multidose drug dispensing bags or a pill organiser. Barriers to medication self-management were a lack of motivation, as they thought medications affected their life too much, e.g. constantly being reminded about medications due to frequent dosing regimens. At the same time, they tried to stay positive and hoped things would improve by sticking to their medication regimen. A few patients were worried when leaving the hospital because they suddenly became fully responsible for the management of medications.

#### 3.2.2 Social interaction strategies

Most patients discussed the importance of investing time in social relationships and staying in contact, especially with family or friends, to facilitate life with MLTCs and medication self-management after hospital discharge. There was less discussion about interactions, relationships, and situations they avoided, but one patient described prioritising family over friends and not having that extra energy. Some patients talked about having to change their approach after hospital discharge to stay in contact, such as phoning more often or inviting people over instead of going out as facilitators. Others described having less contact with people after hospital discharge due to fatigue or being homebound. Some did not seem to have a big network to choose among, which acted as a barrier to medication self-management. The subdomain optimizing social interactions was rarely discussed. The few discussions concerned the importance of socialisation and optimising these to fuel self-management of conditions and medications. These discussions mostly involved patients homebound after hospital discharge who described being dependent on help to get out as a barrier. A facilitator for the optimisation of social interactions mentioned was explaining one's needs.

The patients talked about how disclosing conditions lead to discussions about medication regimens, sometimes comparing medications, exchanging knowledge and experience, and finding peers. At times, sharing information with family and friends was seen as a crucial facilitator for receiving support in managing their medications independently. Overall, patients felt safe and appreciated having someone to disclose information to, thereby gaining understanding. Patients emphasised the importance of timely medication intake, often taking it around others without shame and openly answering any queries. On the other side, some talked about medications as a taboo and barrier to disclosing information (e.g., feeling ashamed or avoidant). One patient described feeling uncomfortable in public places when using a new medication as a barrier to medication self-management.

*Sometimes, I'm not sure if I can inject myself (with insulin) in public places, for example, in a restaurant or something like that. The physician I asked yesterday said it's okay and that I can do it everywhere, but it's kind of uncomfortable in a way. (♀, P3).*

Almost all patients used humour or laughter after hospital discharge to de-dramatize their situation and the changes that had occurred. Some patients used humour as a facilitator to boost motivation for medication self-management after hospital discharge. Several patients laughed about having a hard time remembering the names of all their medications, especially the new medications, but solved this by keeping the packages. One patient had gotten pain medication from a friend after hospital discharge but said, with a glimpse in the eye, she only got one tablet since “*she is afraid she will kill me” (♀, P1)*.

#### 3.2.3 Activities strategies

Many of the patients had started using aids unique to their circumstances or to minimise risks associated with medication-taking (e.g., pill organiser or multidose drug dispensing system) after hospital discharge, facilitating self-management and activities. It seemed more important to use aids, such as pill organisers, when using more medications. Some patients appreciated self-managing their medications by using aids instead of receiving help from HCPs. Barriers for several patients included that they had to wait before receiving needed aids, losing control over their medications when using aids, or that aids increased their stress levels. In addition, some had trouble using aids, such as insufficient finger strength to open a pill organiser.

When asked what they did to manage life with MLTCs and medications after hospital discharge, several patients told they engaged in valued and meaningful activities such as going back to work, studying or hobbies. Some patients used activities as a way of testing how their body worked after the hospital stay. Others had not figured out how to engage in valued activities due to their new situation.

The patients described how they organised routines and systems to facilitate medication self-management and carry out activities after hospital discharge, such as medication lists or reminders such as alarms on the phone. Others incorporated their new medications into a previously established routine. They placed their new medications strategically to remember to take them, for example, in eyesight in the kitchen. Some counted their medications before administering them, removed medications not in use to maintain control or used folders to collect all medical information in one place. Others made sure to replace the old medication list with the new one received at the hospital or took notes of information received at the hospital to facilitate medication self-management after hospital discharge. Some patients seemed to have no system or routines, they simply took some of their medication when they remembered, which was a barrier to medication self-management. One patient received help opening the multidose drug dispensing bags, which made her feel insecure because it interfered with her previous routine.

*Now, I don't know what's in there (in the multidose). I feel very insecure about that, that they should give it to me because what I really like best is that I pick up a package, see what it is, its strength and what it is used for. I take them, and then I take the next package and go through the whole thing. Because, well, it might sound a bit conceited, but I only trust myself (and no one else). (♀, P1).*

Planning one's day in line with the medication taking was frequently discussed. Insecurities about new conditions and associated medications were discussed and patients experienced they must live and learn. One patient had a self-made plan for the period after discharge but expressed a wish for a strict plan from HCPs in the first days after hospital discharge to facilitate medication self-management and guide her into a regimen, e.g., a specified time schedule for taking medications. Several patients talked about the benefit of taking medications at the same time and a few times during the day as a facilitator. Some patients had new medications dosed more frequently than their other medications. These patients described difficulties remembering to bring the new medications to activities, especially in the middle of the day, as barriers to medication self-management. Others planned and brought medications with them when leaving home. Many patients planned and ensured an adequate supply of medications after hospital discharge by going to the pharmacy or using delivery services. Several patients also received medications for a couple of days from the hospital at discharge, which posed as a facilitator for the continuum of medication self-management.

### 3.3 Support-oriented domains

#### 3.3.1 Process strategies

Patients seemed aware of why they took medications, and some had even thought about what they wanted future medication regimens to look like, e.g. stepping down on certain medications. On the other hand, some seemed to have little knowledge about the importance of their medications, lacking any approach to figure it out, which presented as a barrier. One patient had tried tapering down a painkiller after hospital discharge but had to increase the dose again after experiencing severe pain. This patient was aware of choices for pain management and wanted to use another medication. Some patients mentioned the importance of knowledge about side effects and interactions to facilitate medication self-management. A portion of the patients had no methods to increase their knowledge and simply hoped they would be informed, thereby generating barriers.

*I believe that if, for example, I were to stop taking my diabetes medication, things could go really wrong. I also think that the medications related to my heart are necessary to sustain life a while longer. (♂, P10).*

Patients used a combination of verbal and written sources to seek information to facilitate medication self-management, e.g. about medication efficacy or side effects. Some patients preferred printed handouts, asking HCPs, searching the internet or asking friends or family. Several patients thought the multidose drug dispensing system was more informative and safer than pill organisers since the names of the medications, strength and time were printed on the bags.

At the hospital, it was hard for the patients to know what medications they received since they often only got a small cup with pills in it, with no further information. Several patients thought this was a barrier but did nothing, while others thought it was important to ask HCPs questions to strengthen their knowledge to facilitate medication self-management after discharge, such as: “What do I do if I forget to take my medications?”, “Why do I need to take this medication?” or “What is this medication?”. Several patients thought it was hard to remember the loads of verbal information received at the hospital. To be informed of changes affecting medication management, several patients read through the discharge summary or other written information after returning home to facilitate medication self-management. Not all patients received a discharge summary or other written information, expressing a lack of information and barrier to medication self-management, while some were not stressed about not receiving it. Those who received the discharge summary used this to different degrees, e.g. one had continued using the same dosage as before the hospitalisation, even though the summary stated a dose reduction. Some had difficulties understanding medical terms in the discharge summary and medication leaflets, which in turn affected medication self-management negatively. Many patients did not remember anything from the discharge conversation or conversations about medications at the hospital, and some thought a follow-up conversation to get answers to questions they had after hospital discharge would have been helpful. Others had questions after discharge but apparently no methods for seeking information, sometimes due to insufficient digital competence. A few patients described no need to seek information because they received enough information at the hospital to self-manage their medications.

*I've read the package insert (after hospital discharge), and I thought it was strange because I hadn't needed it (the medication) before. It was just whether it could be taken with other tablets because I take so many tablets, especially in the morning. So, I was interested to know if it was dangerous to take them together. And apparently, I shouldn't do that. (♀, P12).*

#### 3.3.2 Resource strategies

Patients discussed how they strategically facilitated medication self-management using various resources for different purposes and when they did not need any resources. Several patients were dependent on support from both unpaid and paid resources, which they had different approaches to. Almost all patients received some kind of unpaid support from family or friends after hospital discharge that supported medication self-management, e.g. collecting medications at the pharmacy, returning deprescribed medications, obtaining information about medications, picking up aids or writing medication lists. The patients expressed the need and appreciation for support from family and friends but valued their autonomy and preferred to self-manage as much as possible. A few patients did not have anyone to ask or did not want to bother those around them, which presented as barriers. Next of kin described not intervening more than necessary and that the patients needed to learn to take responsibility themselves. One next of kin who made a medication list for the patient after discharge believed this eased medication self-management.

*It wasn't difficult for you (the patient) to take them out of the boxes (directly) either, but you just took a little longer. It was also a bit more like, “Oh, how was I supposed to take it again?”. Now, it's a little easier that way (with a medication list). (♀, NK2).*

The patients described how they had sought support from HCPs to facilitate medication self-management after hospital discharge. Several patients had been in touch with HCPs after hospital discharge, some to ask for changes in their medication regimen. Interestingly, some were sceptical and did not trust HCPs completely. After discharge, patients experienced a communication gap between the healthcare levels that acted as a barrier. Patients described having to be the ones informing the next level of care about medication changes because these were not clearly described in the discharge summary. One patient felt insecure about a specific medication in the discharge summary, whether to take it or not, because the summary was unclear.

*I brought it up then, on my first visit to the GP. And then she (the GP) called the hospital to get clarification on that. And the clarification came, and I had to take that medication. (♂, P10).*

Many patients said they never had experienced being involved in shared decision-making, while others said they always were involved. Some patients wished to be more involved in the decision-making processes about medications and said it was important to have the opportunity to state their opinions to facilitate self-management. On the other side, several patients did not want to be involved in such decision-making processes, including setting goals for their treatment and describing barriers such as having enough worries or not having enough knowledge. One patient was prescribed to take 10 tablets of 4 mg of a new medication, even though a higher strength existed. This patient felt no involvement in the decision around this medication at the hospital, even after questioning the hospital staff if it was necessary to take that many tablets. Furthermore, she experienced barriers to medication self-management, such as practical problems fitting all the tablets in the pill organiser.

*There are more tablets to administer, and I think it was very foolish to take 10 (tablets) of that Medrol. I think it was extreme, they also tasted bitter, so I didn't like it, but like now I got 16 (mg) (from the pharmacy), so it worked much better. It makes it easier because each room in the pill organiser isn't that big, so you lose track when you try to count them. (I) wish it was something more than just, for example, it would have helped if there had been slightly different strengths. (I) think that 4 (mg) and 16 (mg) were a bit too low strength (for my needs). (♀, P6).*

## 4 Discussion

The results highlight the complex, interconnected nature of medication self-management by not only addressing the disease-focused aspects of medication use but also situating it within the broader social context of the patient's life, which helps to better understand their individual needs. Our main finding is that facilitators and barriers to medication self-management in the transition from hospital to home are numerous and vary extensively between individuals. To be able to achieve effective medication self-management during care transition and ensure that care is tailored to the individuals' specific needs, lives, and circumstances, it seems important that the facilitators and barriers to medication self-management for each individual are thoroughly understood. Our findings underline the importance of continuously keeping the patient in centre to facilitate medication self-management. There seems to be a need for a change in practice, moving the focus from reactive, visit-based care to a collaborative and preventative patient-centred approach to improve patient satisfaction. This shift has long been discussed in healthcare, as higher patient satisfaction has been linked to improved patient safety, clinical effectiveness, health outcomes, adherence and lower resource utilisation.^31^ Investing in individualisation by keeping the patient at the centre remains a crucial component for delivering high-quality care.^32^^,^^33^

Facilitators and barriers to medication self-management in patients with MLTCs during the transition from hospital to home are as heterogeneous as the patient group itself. Facilitators for medication self-management in the transition from hospital to home were more frequently described than barriers by the participants, indicating that our study participants predominantly have a positive focus on their situation. We found that the two support-oriented domains, i.e. process- and resource strategies, were the most frequently discussed domains. Health behaviour- and social interaction strategies were the least and second least discussed domains, respectively. The one other study using the TEDSS framework with a specific focus on medication self-management found disease controlling strategies to be the most frequent and social interaction strategies to be the least discussed domains.^19^ Differences in what was most discussed may be a result of a different population (persons with spinal cord injury/dysfunction) and setting. In accordance with our findings, other studies have found similar components to facilitate medication self-management, such as support, simplification, acceptance, and the establishment of medication habits, systems, and planning.^34^, 35., 36 The TEDSS framework provided a holistic perspective, encompassing all aspects of medication self-management that might be overlooked by other frameworks focused solely on adherence.^9^ This wide holistic perspective to medication self-management, using TEDSS or other frameworks, has previously been described in other populations and settings.^19^^,^^34^, 35., 36 As shown in our results, these previous studies indicate the importance of assessing all aspects affecting medication self-management to improve overall effectiveness.

The heavy focus on the support-oriented domains in our study underlines how important the patients consider support for medication self-management, which is consistent with previous findings.^19^^,^^36^^,^^37^ Having a good support network was, in our study, identified as an important facilitator to medication self-management. This indicates a need to facilitate support for patients with MLTCs in the vulnerable period after hospital discharge, as earlier implied by Brandberg et al.^13^ Furthermore, the participants described knowing whom to contact, sometimes to control stress, as a facilitator, which is reflected in Brandberg et al.'s research showing that patients with MLTCs were insecure about which HCP was responsible for care after hospital discharge. Several patients in our study expressed a wish for a follow-up conversation to support medication self-management after hospital discharge and make them feel safer. However, these types of follow-up conversations to support medication self-management are generally not an integrated part of patient care.^7^ A previous study described patients reverting to pre-admission routines to medication self-management.^35^ In our study, some previous routines were maintained, but we also found that patients adapted routines and made several changes that affected medication self-management after hospital discharge. Other important facilitators identified included being able to accept conditions and medications to facilitate mastery. Swanlund et al. found that patients valued medications by knowing they were essential to maintain health,^34^ which underlines the importance of strengthening the patient's knowledge about medications.

Important barriers identified included a lack of information, a communication gap between healthcare levels, the burden of taking medications, and not acknowledging the need for all their medications. Lack of information, which has been reported previously,^13^ leads patients to seek HCPs for answers using already scarce healthcare resources. This could be prevented by providing patients with sufficient verbal and written information. In addition, the lack of information could lead to misunderstandings, such as omission of treatment or continuation of discontinued treatment. The current study also highlights the importance of communication between healthcare levels to support medication self-management for patients with MLTCs since patients described having to be the ones informing the next level of care about medication changes, placing the responsibility on the patients. Communication between healthcare levels after a hospital stay is especially important since transitions from hospital to primary care have been well recognised as an area of risk.^38^ Other studies have found comparable results, in that transitions from hospital to primary care are challenging due to missing information about medications, indicating a need for a change in practice around communication.^39^, ^40^, 41. This particularly applies to patients with MLTCs, as they have a higher risk for transfers in the healthcare system due to their range of conditions and complex medication regimens.^3^^,^^4^ Effective communication between HCPs and patients has previously been described as a facilitator to achieve desired health outcomes.^38^

Previous studies have shown that the discharge process is often not tailored to the patient's needs and does not prepare them for post-discharge self-management.^10^^,^^11^ In our study, several patients did not remember if they had a discharge conversation about medications, underlining the importance of empowering patients continuously to increase health literacy, thereby strengthening medication self-management.^42^ In addition, the patients received medications in a cup at the hospital, not always knowing what they got, which could affect medication self-management after hospital discharge when the patients regained responsibility. This unawareness continued after discharge, as the patients talked about how they used their medications, often not in concordance with the discharge summary, making medication self-management even more complicated.

Patients with MLTCs had various barriers to medication self-management within the internal strategies domain, such as describing taking medications as a burden or not seeing the need for all their medications. These barriers were often in synergy with the health behaviour strategies domain and disease controlling strategies domain, e.g., being non-adherent due to non-acceptance. It is known that these patients often experience a treatment burden, as the healthcare system and most treatment guidelines focus on the management of specific conditions alone rather than the whole range of conditions.^1^^,^^4^^,^^43^ These patients often use one or several medications to treat each condition, which might have a negative influence on adherence.^4^ In addition, patients with MLTCs have a high healthcare utilisation and experience fragmentation of care.^1^^,^^4^ It is suggested that the management of MLTCs should focus on supporting patients by maximising self-management potential.^4^ It is evident that support still should be a focus by having a holistic approach adapted to the patient's daily life, increasing care coordination and empowering patients to maximise their self-management potential to avoid hospitalisations.

### 4.1 Strengths and limitations

This qualitative study is one of the first to explore facilitators and barriers to medication self-management for patients with MLTCs in the transition from hospital to home. There are many strengths to this study. The application of the TEDSS framework^18^ led to a broad and increased understanding of facilitators and barriers to medication self-management for patients with MLTCs in the transition from hospital to home. We managed to include patients from several wards and ended up with a heterogeneous sample with respect to demographics such as age, number of medications and assistance with medications, resulting in a broad picture. The interview guide was evaluated and adjusted several times during the data collection period, and the interviews were relatively long, which led to an in-depth understanding of different topics affecting medication self-management.^28^^,^^29^ In addition, we included next of kin where possible and conducted follow-up interviews to enrich the data. The interview guide directed the interviewers into the topics focusing on medication self-management, but the conversation was guided forward by what was said by the participants, maintaining reflexivity.^44^ However, the interviewer's position and prior experiences may have influenced the content of the interviews and possibly the way the data was coded. Nevertheless, the well-defined TEDSS framework helped mitigate these effects, ensuring greater balance and consistency. A relationship with the interviewer was established at the time of inclusion, which could increase the trustworthiness of the findings. In addition, the effect of the setting may have played a role in the trustworthiness of the findings, having almost all patients in their homes and not affected by other than usual surroundings. This has probably enabled the in-depth understanding of the interviewed patients by getting to enter their homes and see how they live as opposed to sitting in an unfamiliar setting. At last, the timing of the interviews reduced the chance of recall bias.

Some limitations to our study should be addressed. The findings are limited to patients with MLTCs transitioning from hospital to home. Furthermore, the heterogeneous sample of patients with MLTCs makes the findings transferable,^44^ but it is possible that the included patients are the most resourceful and more positive in that they brought up more facilitators than barriers. Other types of patients could have resulted in other findings, e.g., including patients with advanced cognitive decline, which may have led to other facilitators and barriers, such as a shift towards more barriers. The study was performed in one country, including patients from three wards at one hospital in Oslo. Cultural or systemic factors might limit the generalisability of the findings, although we strived to include a heterogeneous sample in terms of diversity in demographics, including ethnicity. Most of the transcripts were coded by one author, which may have led to a risk of individual bias influencing the interpretation of the data. However, this potential bias was mitigated during data analysis by collectively reviewing coding performed independently by four of the authors for the first two transcripts. This led to the addition of more descriptions to the codebook and ensured that the coding framework was applied consistently. In addition, another coding meeting was held to maintain analytical consistency.

### 4.2 Recommendations for clinical practice and research

The following recommendations for clinical practice and research are suggested: (1) adopting a holistic approach to support medication self-management tailored to the individual patient's daily life, this requires HCPs to embrace and integrate this perspective when interacting with these patients, (2) based on that philosophy, develop a follow-up program for patients with MLTCs to support safe and effective medication self-management in the transition from hospital to home, (3) investigate if the same facilitators and barriers exist for other patients groups transitioning from hospital to home, and (4) explore facilitators and barriers in other healthcare transitions.

## 5 Conclusion

This study highlights the complex and wide spectre of facilitators and barriers to medication self-management for patients with MLTCs transitioning from hospital to home. Facilitators and barriers to medication self-management were identified in nearly all subdomains of TEDSS, which resulted in numerous and various facilitators and barriers reflecting the complex picture affecting medication self-management in this patient group. Overall, patients focused heavily on facilitators and barriers regarding resources and processes. In clinical practice, patients' medication self-management could be supported through a holistic approach adapted to the individual patient's daily life. This includes improving care coordination and empowering patients to maximise their self-management potential.

## CRediT authorship contribution statement

**Malin Olsen Syversen:** Writing – review & editing, Writing – original draft, Validation, Software, Project administration, Methodology, Investigation, Formal analysis, Data curation, Conceptualization. **Mikas Glatkauskas:** Writing – review & editing, Methodology, Investigation, Data curation. **Liv Mathiesen:** Writing – review & editing, Supervision, Resources, Project administration, Methodology, Formal analysis, Conceptualization. **Marianne Lea:** Writing – review & editing, Supervision, Methodology, Formal analysis, Conceptualization. **Berit Gallefoss Denstad:** Writing – review & editing, Resources, Methodology, Conceptualization. **Karin Svensberg:** Writing – review & editing, Supervision, Resources, Methodology, Formal analysis, Conceptualization.

## Ethical statements

All participants gave written informed consent before inclusion. The participants could at any time withdraw their consent, without any justification. Following consent, the data collectors were given access to the patient's hospital records. The project was approved by the Regional Committee for Medical and Health Research Ethics (Ref No 420920/REK south-eastern C), Sikt (Ref No 919319), and the Privacy Ombudsman at Oslo University Hospital. After the data collection, participants were offered a small gift (5€). The data collectors were not working at the hospital wards. Participation in the study did not pose any risk or harm to the participants beyond the time spent. All data were stored and managed on a secure research server at the University of Oslo.

## Declaration of generative AI and AI-assisted technologies in the writing process

During the preparation of this work the authors used ChatGPT to improve language and rephrase sentences in the manuscript. After using this tool, the authors reviewed and edited the content as needed and takes full responsibility for the content of the published article.

## Funding

This work was funded by the University of Oslo (PhD grant to authors MOS and MG). The funders had no role in the study design, data collection, analysis or interpretation of data, decision to publish, or preparation of the manuscript.

## Declaration of competing interest

The authors declare that they have no known competing financial interests or personal relationships that could have appeared to influence the work reported in this paper.

## Appendix A Supplementary data

Supplementary material 1 Image 1

Supplementary material 2 Image 2

Supplementary material 3 Image 3
