# Supplementary material for: Facilitators and barriers to medication self-management for patients with multiple long-term conditions transitioning from hospital to home
Source: Explor Res Clin Soc Pharm. 2025 Mar 29;18:100598. doi: 10.1016/j.rcsop.2025.100598 (PMC12008552; doi:10.1016/j.rcsop.2025.100598)
Supplement: Supplementary file 1 — Supplementary material 1 [file mmc1.docx]

**Interview guide
Introduction**

- Repetition of aim of study, voluntary participation and the opportunity to withdraw consent. Audiotaping.
- Estimated time frame of the interview, breaks.

**Medication reconciliation performed**

**Self-management**

- If you completely ignore your medications, what do you do to manage/cope with your life or conditions? - What is important to you in life/everyday life? What role do medications have in your life?
- Have you made any changes to your routines after discharge? What about in relation to your medications?
- How do you think the use of medications works for you in everyday life? – How does it affect your life?
- What is your motivation for taking medications? - What practical challenges do you face? What could have been done to make it easier?
- What does it mean to you that you are responsible for the management of your medications?
- What is important to know about new/old medications? Is something less important?
- What do you think is important to be able to manage medications yourself? – How did the hospital facilitate your ability to manage medications when you were discharged? What about your general practitioner (helping you/availability)? Have you experienced receiving different information from the hospital and from the general practitioner? How do you handle the information you receive from healthcare personnel?
- What do you think can increase your quality of life to help you manage daily life with your conditions? What about in relation to medication management? What do you think (does not) work?

**Medication lists**

- How do you gather information about you and your medication treatment? Do the information sources meet your needs? What do you think about the medication information in the discharge summary?
- Which digital solutions do you use to obtain information about your medication treatment/use? What do you think about having access to digital solutions? Are you familiar with HelseNorge? What do you think about it?
- If there was a digital solution that was available to all your healthcare providers and included your medication list, what do you think about that?

**Shared decision making**

- Have you heard about shared decision making? What does shared decision making mean to you? How important is it for you to be involved in decisions about what kind of medications you should use? Who should determine the goals of your treatment? How do you involve in your own medication use?
- Have you previously been presented with alternatives to your medication treatment? Do you have examples? What information did you recieve about the various options? How do you think the communication is adapted to you? Do you receive written information?
- How do you feel you are involved in decisions made regarding your medication treatment? E.g. at the hospital or with the general practitioner? What do you feel limits the possibilities of taking responsibility yourself? What do you think build your trust in healthcare personnel?

**End of interview**

- Is there anything you as a medication user could suggest that would have made medication use easier, more understandable, or just better for you or others?
- Something more to add?
- Opportunity to stay in touch, e.g. to add or request more information.
- Thank you so much for your time and a small gift.
