# Supplementary material for: Facilitators and barriers to medication self-management for patients with multiple long-term conditions transitioning from hospital to home
Source: Explor Res Clin Soc Pharm. 2025 Mar 29;18:100598. doi: 10.1016/j.rcsop.2025.100598 (PMC12008552; doi:10.1016/j.rcsop.2025.100598)
Supplement: Supplementary file 2 — Supplementary material 2 [file mmc2.docx]

Codebook

Codebook used in our study with domains and subdomains in The Taxonomy of Every Day Self-management Strategies (TEDSS) framework including description.^1^ Domains are marked with bold text font. More description was added to fit our study focusing on medication self-management.^2^ TEDSS subdomain data were sorted into positive (+) or negative (-) categories to identify facilitators and barriers.

| Domains and Subdomains | Description | More description |
| --- | --- | --- |
| **Disease controlling strategies** | Preventing, controlling and limiting symptoms, complications and/or disease progression |  |
| Manage medication and treatments | Taking prescribed medication and/or over the counter medication. Completing treatments at home. | For example deciding to take, not to take, completely stop medications or to change regimen because of side effects, personal preferences, non-medication alternatives, lifestyle choices (vacation) |
| Prevent symptoms and complications | Accommodating and controlling symptoms and disease related complications (limiting the risk of falling, having an annual flu shot, avoiding seizure triggers) and controlling/limiting existing symptoms (stretching, wound care, hot packs to limit pain). | Includes monitoring/controlling and managing side-effects. Also includes taking medications or using medical equipment as needed to control symptoms (e.g. pain, fever, cold/flu). Management of secondary complications. |
| Use complementary medicine | Using supplements (e.g., herbal remedies, probiotics etc.) and complementary strategies (massage, conductive education) | In addition for example vitamins/minerals, marijuana, acupuncture, physiotherapy, music therapy, chiropractic care, psychology |
| **Health behavior strategies** | Maintaining a healthy lifestyle in order to enhance health and limit the risk of lifestyle related illness. |  |
| Diet | Maintaining healthy eating habits. Eating more healthy foods (e.g. vegetables, fruit, protein, water consumption, vitamins) and avoiding unhealthy foods (e.g. sweets, deserts, processed foods). | For example food as source of vitamins, moderating diet as alternative to avoid medications |
| Mental exercise | Keeping mentally fit (e.g. brain teasers, games, puzzles, committee membership or volunteering). | In addition also for example reading books |
| Physical exercise | Being physical active (e.g. sports, gym exercise, walking, therapeutic stretching, or swimming) within level of functional ability. | In addition also for example physiotherapy, aqua therapy |
| Sleep hygiene | Creating a healthy sleep routine, including regular bedtime, calm activities before sleeping, attention to mattress and pillow, and use of needed, routine daily naps. |  |
| **Internal strategies** | Preventing and managing stress, negative emotions and internal distress; creating inner calm. |  |
| Acceptance | Accepting issues and conditions judged to be out of one’s control and gaining inner peace with unchangeable circumstances. | For example coming to terms with/accept the use of medications/conditions/preventative strategies, sometimes with much resignation |
| Allowing time for sadness and grief | Expressing feeling (e.g. crying or venting) with the intention to feel better afterwards. |  |
| Controlling stress and negative emotions | Controlling emotions in order to remain calm, reduce anxiety and/or prevent being overwhelmed by emotions. Using techniques like meditation, breathing techniques and relaxation, or deliberately avoiding thinking about problems, symptoms or future risks by focusing on other activities or thoughts. | For example being proactive to avoid possible negative situations |
| Seeking comfort in faith and spirituality | Praying, talking with religious leaders or reading spiritual texts, in order to feel inner comfort. |  |
| Staying positive | Deliberately adopting a positive attitude to limit negative feelings and generate positivity. Reevaluating one’s situation, finding meaning and perspective in life, sometimes to fight depression. | For example having a good attitude towards life post discharge/conditions and staying motivated, impact of side effects etc. on the ability to stay positive |
| **Social interaction strategies** | Managing social interactions and relationships to be able to participate without exposure to negative reactions |  |
| Choosing social relationships and situations | Prioritizing and investing in selected interpersonal relationships. Avoiding emotionally demanding or discriminating interactions; ensuring a sense of control. | For example the importance of staying in touch with family/friends |
| Disclose condition | Deciding to whom to disclose information about condition, including what and how much information is given to each person. |  |
| Optimize social interactions | Facilitating interactions (talking slowly, rephrasing sentences, explaining needs). Controlling misunderstood symptoms (e.g. spasms or drooling), or emotions (e.g. anger or anxiety). | For example the importance of socialization, including with family |
| Stay in contact | Staying in contact with family or friends using traditional and new methods of connecting, sometimes to overcome cognitive or mobility problems (e.g. using social media when home bound). |  |
| Use Humor | Using humor or laughter to de-dramatize a situation. |  |
| **Activities strategies** | Finding ways to participate in everyday activities (leisure activities, work activities, household chores) despite problems such as fatigue, pain, memory loss or disability. |  |
| Aids and physical adaptations | Using aids (e.g. canes, mug with straw), adapting environments (e.g. rearranging furniture to ease movement, ramps) and adapting behaviors (e.g. having a hand on the wall while moving around) to facilitate activities. | For example wheelchairs, blister packs, medication cups, adapting bottles/pill organisers, unique to patients’ circumstances or to minimize risks associated with medication-taking. |
| Engage in valued activities | Making time to do the activities that are important and bring meaning and value to the individual. For example being with family, taking a walk, painting, attending a concert. | For example spending time with friends/family, going to work, physical therapy, finance, study, leisure activities, socialization. |
| Organizing routines and systems | Using tables, charts, lists, reminders, tracking systems and routines to organize information, items and equipment and to carry out activities. | For example daily planners, calendar/schedules, alarms, app, smart phone, routine, pill organisers, blister packs, taking notes/making lists. Includes ways to take medicines. |
| Pace, plan and prioritize | Using time wisely, planning the day, resting to conserve energy, adapting activities to current functional level and making important activities a priority. | For example planning ahead to ensure adequate medication supply, prioritizing daily routines around medication-taking, planning day around medication-taking. |
| **Process strategies** | Strategies used to be well informed and to make good decisions. Often used to support use of other, non-process strategies. |  |
| Awareness and problem-solving | Proactive strategies to become aware of choices and consequences of disease related problems in everyday life, trying to find alternative solutions and making informed decisions. | For example knowledge about their bodies/medications/conditions, fitting medications into lifestyle, side effects, secondary complications, ensuring supply of medications. |
| Information seeking | Researching and seeking information regarding, for example, one’s disease, symptoms and treatment, living with illness, health, social service insurance systems. | For example from healthcare providers, online, research articles, peers, google, websites, printed handouts. |
| **Resource strategies** | Proactively seeking, pursuing and/or managing needed formal or informal supports and resources. |  |
| Seeking and managing everyday support | Judging the need for support, asking for support, planning support, and keeping a sense of autonomy despite receiving support. | For example seeking information about alternatives/assistance with medication-taking, support for everyday activities, having a caregiver that can provide support (e.g. administering medications, obtaining information). This category refers to others than health care personnel. |
| Seeking and managing health/social-care needs and paid support | Navigating and managing the formal support health-care systems (health, social, financial) in order to receive treatment, referral, equipment, etc. Includes seeking health care, attending appointments and preparing oneself for consultations. | For example doctors, pharmacists, specialists, attendants, personal support workers, occupational therapists, home and community care nurses. Seeking support for information about medications, pain management, overall health |
| Self-advocating | Actively pursuing access to healthcare providers, social systems, and legal rights. Speaking out against discrimination. | For example reaching out to healthcare providers to make changes, involved/present in decision making processes, advocate for drug coverage or the use of natural health products etc. |

**References**

1. Audulv, Å., et al., *The Taxonomy of Everyday Self-management Strategies (TEDSS): A framework derived from the literature and refined using empirical data.* Patient Educ Couns, 2019. **102**(2): p. 367-375.

2. Cadel, L., et al., *Exploring the perspectives on medication self-management among persons with spinal cord injury/dysfunction and providers.* Res Social Adm Pharm, 2020. **16**(12): p. 1775-1784.
