# Supplementary material for: Facilitators and barriers to medication self-management for patients with multiple long-term conditions transitioning from hospital to home
Source: Explor Res Clin Soc Pharm. 2025 Mar 29;18:100598. doi: 10.1016/j.rcsop.2025.100598 (PMC12008552; doi:10.1016/j.rcsop.2025.100598)
Supplement: Supplementary file 3 — Supplementary material 3 [file mmc3.docx]

Facilitators and barriers
The Taxonomy of Every Day Self-management Strategies (TEDSS) domains and subdomains^1^ with examples of facilitators and barriers for each subdomain.

| *Domain* | *Subdomain* | *Facilitators* | *Barriers* |
| --- | --- | --- | --- |
| **Disease controlling strategies** | **Manage medication and treatments** | *● Knowledge about the medication’s benefits*  *● Motivated to get back to normal* | *● Unaware of medication changes*  *● Non-adherence due to personal preferences* |
|  | **Prevent symptoms and complications** | *● Availability of medications at home ● Knowing when and why to take medications* | *● Not knowing how to handle symptoms ● Not fan of using medications* |
|  | **Use complementary medicine** | *● Knowing complementary medicine supplement medications ● Important for management of conditions* | *● Scepticism and lack of belief ● Less important to incorporate into daily routines than their medications* |
| **Health behaviour strategies** | **Diet** | *● Knowledge about food-medication interactions ● Using food to avoid the need for medications* | *● Differences between hospital food and what is normally eaten at home affecting medication self-management ● Remember to take certain medications before meals* |
|  | **Mental exercise** | *● Using mental exercise to support medication self-management ● Having someone or something to be mentally challenged* | *● Unaware of the benefits of mental exercise ● Not taking time to be mentally challenged* |
|  | **Physical exercise** | *● Using physical exercise to be independent* *● Fuel motivation to stop or avoid medications* | *● Lacking motivation  ● Fees for physiotherapy  ● Insufficient pain relief* |
|  | **Sleep hygiene** | *● Regular bedtime or calming activities to avoid sleep medication*  *● Having a medication regimen positively affecting sleep hygiene* | *● New medication regimens affecting sleep routines*  *● Not regaining routines after hospital discharge* |
| **Internal strategies** | **Acceptance** | *● Accepting conditions and medications to facilitate mastery ● Accepting help to avoid medication errors* | *● Burden of taking medications*  *● Not seeing the need for all their medications* |
|  | **Allowing time for sadness and grief** | *● Allowing to relieve stress and feeling better afterwards ● Looking forward on what they can do about their situation* | *● Not having enough pain medications causing negative feelings ● Looking back and seeking comfort in old habits* |
|  | **Controlling stress and negative emotions** | *● Focus on positive things and avoid thinking about problems  ● Knowing whom to contact ● Techniques to avoid medications* | *● Focusing on the negative things ● Not having any methods to cope with stress or negative emotions* |
|  | **Seeking comfort in faith and spirituality** | *N.A.* | *N.A.* |
|  | **Stay positive** | *● Positive to using medications to get better ● Sticking to medication regimen and looking forward* | *● Medications affecting life to much ● Suddenly having full responsibility of medications* |
| **Social interaction strategies** | **Choosing social relationships and situations** | *● Investing time with family or friends to fuel medication self-management ● Prioritizing social relations and situations due to the new situation* | *● Not having the ability or network to choose relationships or situations* |
|  | **Disclose condition** | *● Necessary to receive support and understanding ● Disclosing information leading to finding peers and knowledge exchange* | *● Thinking medications is a taboo, thus not disclosing information ● Feeling uncomfortable, ashamed or avoidant* |
|  | **Optimize social interactions** | *● Telling others what you need ● Optimize to fuel self-management of conditions and medications* | *● Dependent on help to socialize* |
|  | **Stay in contact** | *● Finding new methods to stay in contact ● Staying in contact to facilitate life with MLTCs and medication self-management* | *● New conditions leading to less contact and missing out* |
|  | **Use humour** | *● Using laughter when not remembering medication names ● De-dramatize a situation with humour* | *N.A.* |
| **Activities strategies** | **Aids and physical adaptations** | *● Pill organisers or multidose drug dispensing bags when using more medications ● Aids facilitating for self-management and activities* | *● Long waiting time for aids ● Aids not adapted to the patients’ circumstances* |
|  | **Engage in valued activities** | *● To manage life with conditions and medications ● A way of testing their body* | *● Not knowing how to engage due to their new situation* |
|  | **Organizing routines and systems** | *● Medication lists or alarms to ease medication self-management ● Removing medications not in use* | *● Sudden changes in medication routines ● Not having a manageable system* |
|  | **Pace, plan and prioritize** | *● Strict plan from HCP in the first days after hospital discharge ● Taking medications at the same time and few times during the day ● Plan and ensure adequate medication supply* | *● Not having anything to lean on, not knowing if the self-made plan is good enough ● Being prescribed new medications with more frequent dosing* |
| **Process strategies** | **Awareness and problem-solving** | *● Knowing why they take medications ● Knowledge about options of medications* | *● No knowledge about the importance of their medications ● Hoping to be told about medications side-effects or interactions* |
|  | **Information seeking** | *● Combination of verbal and written information sources ● Asking questions about medications ● Follow-up conversation with HCP* | *● Not invested enough to seek information ● Not receiving a discharge summary or information to support medication self-management ● Incomprehensible medical terms ● Insufficient digital competence* |
| **Resource strategies** | **Seeking and managing everyday support** | *● Support tasks associated with medication self-management ● Maintaining autonomy despite receiving support* | *● Not having friends or family to ask for help ● Not wanting to bother others* |
|  | **Seeking and managing health/social-care needs and paid support** | *● Trustful relationship to HCP ● Seeking HCP if uncertainties occur* | *● Communication gap between healthcare levels ● Navigating and understanding the healthcare system* |
|  | **Self-advocating** | *● Speak up about what you want and need of medications ● HCPs using a language that support patient involvement and being open to patients’ opinions* | *● Not empowered enough to involve in decision making ● Not familiar with the concept of shared decision making ● Reluctant to be involved or take responsibility* |

Abbreviations: HCP, healthcare personnel, MLTCs, multiple long-term conditions.

**References**

1. Audulv, Å., et al., *The Taxonomy of Everyday Self-management Strategies (TEDSS): A framework derived from the literature and refined using empirical data.* Patient Educ Couns, 2019. **102**(2): p. 367-375.
